# Systematic review: total knee arthroplasty (TKA) in patients with pigmented villonodular synovitis (PVNS)

**DOI:** 10.1186/s43019-021-00088-1

**Published:** 2021-02-25

**Authors:** Yi Chuen Tan, Jia Yin Tan, Konstantinos Tsitskaris

**Affiliations:** 1 Whipps Cross University Hospital, Barts Health NHS Trust, Whipps Cross Road, Leytonstone, London, E11 1NR UK; 2 John Radcliffe Hospital, Oxford University Hospitals NHS Foundation Trust, Headley Way, Headington, Oxford, OX3 9DU UK; 3 Orthopaedics Department, Whipps Cross University Hospital, Barts Health NHS Trust, Whipps Cross Road, Leytonstone, London, E11 1NR UK

**Keywords:** Pigmented villonodular synovitis, Total knee arthroplasty, Functional outcomes, Complications

## Abstract

**Background:**

To determine the functional outcomes, complications and revision rates following total knee arthroplasty (TKA) in patients with pigmented villonodular synovitis (PVNS).

**Materials and methods:**

We conducted a systematic review of the literature. Five studies with a total of 552 TKAs were included for analysis. The methodological quality of the articles was evaluated using the Strengthening the Reporting of Observational Studies in Epidemiology (STROBE) scale. Functional outcomes, complications and revision rates were assessed. The mean age was 61 years (range 33–94 years) and the mean follow-up period was 61.1 months (range 0.2–35 years).

**Results:**

All the studies reported improvement in knee function following TKA. Post-operative stiffness was the most frequently reported complication, affecting 32.7% (*n* = 32) of patients in our review. Symptomatic recurrence of PVNS, component loosening, tibial-component fracture, instability and periprosthetic infection were the main factors leading to the need for revision TKA.

**Conclusion:**

The findings of this review support the use of TKA to alleviate the functional limitations and pain due to knee degeneration in patients with PVNS. The operating surgeon should be aware of the increased risk of post-operative stiffness, as well as a potentially higher risk of infection. Implant survival should also be considered inferior to the one expected for the general population undergoing TKA.

## Introduction

Pigmented villonodular synovitis (PVNS) is a benign proliferative condition which affects the synovial tissue and is a subtype of tenosynovial giant-cell tumor [1]. This rare condition is believed to be an inflammatory process and can either be localized (L-PVNS) or diffuse (D-PVNS), the latter accounting for the majority of cases [2]. PVNS commonly presents unilaterally in the knee, hip and ankle joints, with the diffuse form typically characterized by joint pain, swelling and stiffness, while the localized form presents with locking, catching and joint instability. The disease typically affects individuals aged 30–40 years old but can affect patients in all age groups [3].

The etiology of PVNS is still unclear, with some believing that the disease stems from chronic inflammation [4, 5], whereas others classify it as a benign tumor with low metastasizing potential [6, 7]. Trauma has also been implicated in the development of PVNS, but with poor evidence supporting a causal link between the two.

Treatment for PVNS aims at removing the pathological lesion(s) either through open or arthroscopic synovectomy [8]. A meta-analysis by Aurégan et al. suggests that there were no significant differences between open or arthroscopic synovectomy when it came to functional outcome and local recurrence in both patients with L-PVNS and D-PVNS of the knee; however, in D-PVNS, there were fewer post-operative complications when performing arthroscopic synovectomy [9]. Another meta-analysis conducted by Mollon et al. [10] suggests that open synovectomy or synovectomy combined with peri-operative radiotherapy for D-PVNS is associated with a reduced rate of recurrence and has good functional outcomes; however, the authors note that open synovectomy also brings an increased risk of stiffness when compared to arthroscopic synovectomy [10]. In the setting of PVNS with established degenerative changes, total knee arthroplasty (TKA) should be considered [9].

With the increasing number of TKAs performed each year, arthroplasty surgeons are likely to encounter cases of PVNS with established degenerative changes. Understanding the challenges associated with this group of patients is, hence, very important. Our initial intent was to perform a meta-analysis on TKA as a treatment modality for PVNS; however, due to both the disease’s rarity and the scarcity of available literature, we have, therefore, decided to perform a systematic review of the literature instead to determine the functional outcomes and complications of TKA in patients with PVNS of the knee.

## Materials and methods

### Search strategy

The literature search was conducted on Medline and EMBASE on 16 April 2020 by research librarians at two independent hospitals. Keywords used for the searches were “Pigmented Villonodular Synovitis” OR “Pigmented Villonodular Tenosynovitis” OR “Giant Cell Tumour of Tendon Sheath” AND “Arthroplasty, Replacement, Knee.” All relevant studies between 1946 and 2020 were identified in accordance with the Preferred Reporting Items for Systematic review and Meta-Analysis (PRISMA) guidelines. All three authors then identified relevant studies by assessing the bibliographies of the included papers. The selected articles adhered to the PICO criteria for systematic reviews Population, Intervention, Comparison and Outcomes (PICO) criteria for systematic reviews.

### Eligibility criteria

Our inclusion criteria for the study were as follows: (1) the studies selected should be about TKA as a treatment modality in PVNS, (2) the articles should be written in English, (3) the articles would report the functional outcomes post TKA and (4) the articles would report on complications post TKA.

Our exclusion criteria were: (1) articles not reporting on the functional outcomes, (2) articles which included arthroscopic treatment of PVNS instead of TKA and (3) case reports which did not include the functional outcomes post TKA.

After removal of duplicate articles, a full-text review of the selected studies was undertaken by two authors (YCT and KT).

### Data extraction

One reviewer (YCT) extracted data from the selected papers using a standardized data collection form. Information relating to the number of patients, their demographics, follow-up period, complications, revision rates, implant survival rates, recurrence and pre-operative and post-operative clinical and functional outcomes were compiled into a spreadsheet which was later checked by the second reviewer (JYT). There were no inconsistencies in the results.

### Quality assessment

The Strengthening the Reporting of Observational Studies in Epidemiology (STROBE) scale was used to evaluate the articles [11]. It was performed independently by both reviewers (YCT and JYT). This scale comprised a 22-item checklist that assesses the title, abstract, introduction, methods, results, discussion and other information in an article. It was then divided into 34 individual items, for which each item received a score of 0 to 1. Each article was scored out of a total score of 34. The scores were compared between both reviewers and discrepancies discussed until consensus was reached. The total score was converted to represent a percentage form. Evaluation of quality assessment was classified into three categories, with more than 80% of criteria met = ++, 50 to 80% of criteria met = + and less than 50% of criteria met = −.

The four studies included were small to medium retrospective case series (*n* = 10–48 patients, 11–48 TKAs) describing the outcome of TKA in patients with PVNS. Our largest study was by Casp et al. (*n* = 453 TKAs) [12] and we decided to include this study as it was a large study that reported on the complications after TKA in patients with PVNS even though the study did not report on the pre-operative and post-operative functional outcomes. The range of follow-up in these studies was from 0.2 to 35 years. Using the STROBE checklist, the four articles by Houdek et al. [13], Casp et al. [12], Lei et al. [14] and Su et al. [15] scored a maximum score of 100%. The article by Verspoor et al. [16] scored 97% as there was no usage of sensitivity analyses. However, this article was still included as it reported relevant information on recurrence and complication of TKA. The quality scores based on the percentage of the STROBE checklist fulfilment for each article are illustrated in Table 1.

**Table 1:** Studies included, study size, Knee Society Score (KSS), range of movement (ROM), stiffness, recurrence and quality assessment

Author	Year of study	Study size	KSS (functional)	KSS (clinical)	ROM pre-op	ROM post-op	Mean ROM (pre-op)	Mean ROM (post-op)	Stiffness	Recurrence	Quality assessment^**a**^
Houdek et al. [13]	2017	48 TKAs D-PVNS: 40 L-PVNS: 8	45 to 62	54 to 87	30–35^o^	60–130^o^	99^o^	101^o^	13 knees	6 knees	++
Su et al. [15]	2019	28 TKAs D-PVNS: 28 L-PVNS: 0	48.9 to 84.6	38.9 to 84.4	60–100^o^	90–130^o^	86.1^o^	107^o^	0 knees	0 knees	++
Verspoor et al. [16]	2016	12 TKAs D-PVNS: 8 L-PVNS: 4	70.45 (post-op only)	77.6 (post-op only)	NR	NR	NR	NR	1 knee	1 knee	++
Lei et al. [14]	2016	11 TKAs D-PVNS: 8 L-PVNS: 3	35.0 to 81.8	40.5 to 90	60–100^o^	95–120^o^	80.5^o^	109.5^o^	0 knees	0 knee	++
Casp et al. [12]	2018	453 TKAs	NR	NR	NR	NR	NR	NR	31 knees	NR	++
		**Total:** **99 TKAs (552 TKAs including Casp et al.)** **D-PVNS: 84** **L-PVNS: 15**	**74.71**	**84.75**			**92.51**^**o**^	**104.01**^**o**^	**45 knees**	**7 knees**	

^a^Quality assessment of included articles (percentage of Strengthening the Reporting of Observational Studies in Epidemiology (STROBE) checklist met, < 50% = −, 50–80% = +, > 80% = ++). The STROBE checklist is available at https://www.strobe-statement.org/index.php?id=available-checklists

*TKA* total knee replacement, *D-PVNS* diffuse pigmented villonodular synovitis, *KSS* Knee Society Score, *L-PVNS* localized pigmented villonodular synovitis, *NR* not reported, *pre-op* pre-operative, *post-op* post-operative, *ROM* range of motion

### Statistical analysis

The results were summarized using descriptive statistics for continuous variables, frequencies and percentages for categorical variables. Microsoft Excel, 2016 version (Microsoft Corporation, Redmond, WA, USA) was used for data analysis.

## Results

### Search results

**Fig. 1:**
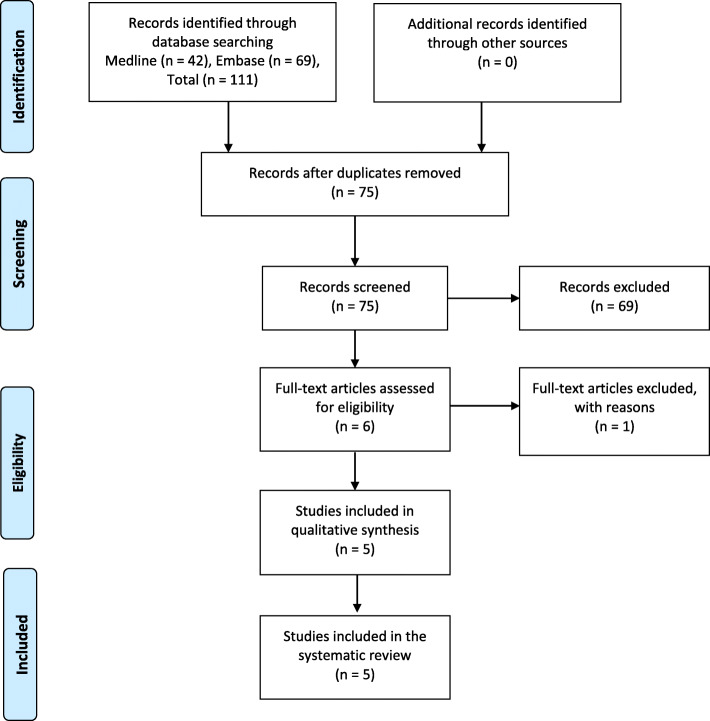
Preferred Reporting Items for Systematic review and Meta-Analysis (PRISMA) chart

The initial literature search identified 111 articles, of which 36 were duplicated articles. After screening the remaining 75 articles, a total of six studies satisfied the eligibility criteria. Two articles, Houdek et al. [13] and Hamlin et al. [17], were published by the same organization 11 years apart. We contacted the authors to clarify whether there was any data overlap, but they were unable to confirm or deny this. We, thus, elected to omit the article by Hamlin et al. [17] as it reported on the smaller series. In the end, five studies were included in our data extraction. All five studies included post-operative complications, four studies included the functional outcome in terms of Knee Society Score (KSS) and post-operative range of motion (ROM) and three studies included the pre-operative ROM. Figure 1 outlines the search strategy.

### Cohort characteristics

The studies included 552 TKAs; however, only 98 patients (99 TKAs) had reported functional outcomes. Among the 552 TKAs, 83 patients (84 TKAs) had D-PVNS and 15 had L-PVNS, while the remaining 453 patients from the study by Casp et al. were not characterized by the disease subtype [12]. The mean patient age of the four studies was 61 years (range 33–94). Authors Casp et al. [12] did not provide a mean patient age and only included a range of ages in their study. Overall, the mean follow-up period was 61.1 months (range 0.2–35 years). Again, as Casp et al. [12] did not provide a mean age and follow-up period, this was omitted from the results above.

### Outcome analysis

#### Functional outcome

Three studies reported a pre-operative and post-operative comparison of the KSS and demonstrated a mean improvement of 26.79 and 39.11 points post-operatively, in functional and clinical KSS respectively. Casp et al. [12] and Verspoor et al. [16] did not provide the pre-operative KSS.

#### Pre-operative and post-operative range of motion

Both studies by Lei et al. [14] and Su et al. [15] showed an average of a 32° improvement in post-operative ROM. Houdek et al. [13] reported no significant changes in the pre-operative and post-operative ROM (*p* = 0.72). The study by Casp et al. [12] and Verspoor et al. [16] did not report on the ROM.

#### Stiffness

Stiffness or decreased knee range was defined by authors Houdek et al. [13] as a flexion contracture of ≥ 15° or flexion < 90°. There were 35 cases (6.3%) of stiffness within the 1-year post-operative period among the five studies included. The study by Casp et al. [12] utilized the current procedural terminology (CPT) coding system to identify patients who underwent TKA and had a diagnosis of PVNS or a procedure for stiffness within 1 year post-operatively. They noted a significant rate of stiffness in the PVNS group of 6.84% (*n* = 31 patients) compared to 4.69% (*n* = 85 patients) in the control group with osteoarthritis (odds ratio (OR) 1.48, *p* value = 0.023).

#### Infection

In total, there were a total of 16 infections (2.9%) in our patient population. Authors Casp et al. [12] utilized CPT codes to identify any cases of infection, while authors Houdek et al. [13] identified infection during their follow-up period. The remaining three authors reported that there were no infections noted during the follow-up period.

#### Recurrence

“Symptoms of disease recurrence” was defined by authors Houdek et al. [13] as recurrent effusions, lytic formations and hemarthrosis. Authors Su et al. [15] defined it as worsening pain with or without a decrease in ROM or the finding of a new palpable mass. All patients included in the study were followed up in clinic and were monitored for disease recurrence. Houdek et al. [13] utilized plain radiography to diagnose recurrence, Verspoor et al. [16] utilized joint ultrasound and Su et al. [15] utilized magnetic resonance imaging (MRI). Excluding the Casp et al. [17] paper, which did not report on recurrence rates, the recurrence rate following TKA in our review was 7.1% (seven patients), six patients from the Houdek et al. [13] study and one patient from the Verspoor et al. study [16]; with a mean of 6 years (range 2–12 years). All seven patients who had recurrence had active D-PVNS. There were no reported cases of recurrence in L-PVNS.

#### Revision rate

Revision was described as the removal of implants with or without replacement of the materials. Symptomatic recurrence of PVNS, component loosening, tibial-component fracture, instability and periprosthetic infection were the main factors leading to revision TKA [13]. There was a total of 22 patients who underwent revision surgery in the five studies. The study by Casp et al. [12] reported that 11 patients underwent revision surgery post TKA; however, they noted that there was no significance (*p* value = 0.92) in increased risk of revision at 2 years compared to the osteoarthritis (OA) group in the 2 years post-operative period. Authors Verspoor et al. [16] reported that one patient underwent revision surgery 6 years post TKA for medial tibial-component loosening. Authors Houdek et al. [13] reported a total of 10 patients, with four patients having disease recurrence, three patients with component loosening and osteolysis, and the remaining indications were tibial-component fracture, instability and deep infection. Authors Lei et al. and Su et al. did not report on revision rates in their studies [14, 15].

## Discussion

TKA has been shown to be a generally successful procedure in patients with PVNS and established degenerative changes and this is reflected in the improvement of post-operative functional and clinical KSS in the four studies included in this systematic review. The main complication of PVNS in our patient cohort appears to be stiffness and infection.

A systematic review and meta-analysis of case series and national registry reports with more than 15 years of follow-up by Evans et al. [18] estimated that the 25-year survival rate of TKA was 82%. The study also included an analysis done by the UK Clinical Practice Research Datalink (CPRD) in 2017 which estimates that the survival rate of TKA is 89.7% (95% confidence interval (CI) 87.5–91.5) at 20 years [18]. In comparison, implant survival rate of patients with PVNS was reported by Houdek et al. [13] to be 89% at 5 years, 80% at 10 years and 62% at 20 years. The other authors did not report on the implant survival rates.

An interesting observation from the studies was that 33 patients (34 TKAs) were diagnosed with PVNS post-operatively, through histopathological samples taken during the arthroplasty procedure due to suspicious synovial changes. This was seen in 23 out of 28 patients (82.1%) in the Su et al. study [15] and 10 out 10 patients (100%) in the Lei et al. study [14] and reinforces the message that intraoperative findings of synovial proliferation and reddish-brown, pigment-stained, synovial tissue should alert the operating surgeon to take tissue biopsies.

Stiffness was among the main complications post TKA in patient with PVNS. According to the UK National Joint Registry 16th Annual Report, the peak incidence for stiffness occurs between 1 and 3 years with 0.56 revisions performed per 1000 primary knee replacements (95% CI 0.53–0.60) with the trend for the development of stiffness decreasing after the 3-year mark [19]. There were 35 (6.3%) patients who presented with stiffness within 1 year post-operatively among the five studies in this review. Houdek et al. [13] highlighted that the group of patients who had undergone previous open synovectomy for PVNS had poor pre-operative knee ROM (mean 78^o^, range 50–115^o^) which co-related with their poor post-operative ROM; however, there appears to be no clear link between multiple interventions and increased stiffness post TKA in our patient population. Casp et al. [12] evaluated knee PVNS as a risk factor for complication after TKA. They compared patients who had undergone a TKA for knee degeneration with PVNS versus a control group consisting of patients with knee osteoarthritis and found that the PVNS group had a higher incidence of post-operative stiffness at 1 year (6.84% versus 4.69%, OR 1.73, *p* value = 0.011).

There was a total of 16 infected joints in our patient population. Casp et al. [12] also compared the risk of infection between the group with PVNS and the group with osteoarthritis and reported an infection rate of 3.31% (*n* = 14) in the former versus 1.55% in the latter (OR 1.73, 95% CI 1.31–2.30, *p* value = 0.011). Houdek et al. [13] reported a single case of periprosthetic infection which was eventually addressed with revision TKA. This brings the infection rate up to 2.9% for this review. Both Lei et al. [14] and Su et al. [15] administered a cephalosporin-based antibiotic post-operatively and had no infections in their follow-up, indicating that there may be a role for extended antibiotics prophylaxis in preventing infections. However, given the very small number of patients in these studies, this claim warrants further investigations.

A total of seven patients were noted to have a recurrence of PVNS at a mean of 6 years (range 2–12 years) following the TKA. Both Houdek et al. [13] and Verspoor et al. [16] reported an association between previous surgery and the diffuse variant, with recurrence of PVNS post-operatively. It is worth noting that recurrence is a time-dependent phenomenon, and its incidence may increase with longer follow-up. It is also associated with the extent of the synovectomy performed during TKA and it is widely considered that total synovectomy during the TKA is critical in preventing recurrence of PVNS [14, 20]. Mollon et al. [10] performed a systematic review of surgical synovectomy in the treatment of PVNS and reported that open synovectomy or synovectomy combined with peri-operative radiotherapy is associated with a reduced rate of recurrence of PVNS. Post-operative radiotherapy could, hence, be considered as an adjunct to synovectomy following TKA for PVNS, with a view to further suppressing the incidence of recurrence.

Post-operative recurrence of PVNS can be identified with MRI, using metal artefact reduction sequence that optimizes the visualization of periprosthetic soft tissues. MRI can play an important role during post-operative surveillance as it is highly sensitive and non-invasive. Relevant findings are a mass or diffuse synovitis of low signal intensity, both of which warrant close follow-up [21].

### Limitations

This systematic review has limitations. First, we have reported the pertinent complications, but recognize that there is a time-dependent bias, which, due to the relatively short follow-up, may lead to underrepresentation of their true incidence. Second, the available studies were characterized by having a low level of evidence as well as a lack of a complete uniformity in reporting outcomes. Additionally, the Casp et al. [12] study did not look at the functional outcome of TKA in PVNS; however, we have included it in our review as the paper was a large study looking at the complications post TKA in patients with PVNS. Lastly, due to disease rarity, there is a lack of literature available on TKA in PVNS. As a result, a meta-analysis was not performed.

## Conclusion

The findings of this review support the use of TKA to alleviate the functional limitations and pain due to knee degeneration in patients with PVNS. The operating surgeon should be aware of the increased risk of post-operative stiffness, as well as a potentially higher risk of infection. Implant survival should also be considered inferior to the one expected for the general population undergoing TKA.

## Data Availability

All data generated or analyzed during this study are included in this published article.

## Abbreviations

PVNS: Pigmented villonodular synovitis
L-PVNS: Localized pigmented villonodular synovitis
D-PVNS: Diffuse pigmented villonodular synovitis
TKA: Total knee arthroplasty
PICO criteria for systematic reviews: Population, Intervention, Comparison and Outcomes
ROM: Range of motion
KSS: Knee Society Score
CPRD: Clinical Practice Research Datalink
STROBE: Strengthening the Reporting of Observational Studies in Epidemiology
